# Glial phenotype modulators

**DOI:** 10.18632/oncotarget.16245

**Published:** 2017-03-15

**Authors:** Kyoungho Suk

**Affiliations:** Department of Pharmacology, Brain Science & Engineering Institute, BK21 Plus KNU Biomedical Convergence Program, Kyungpook National University School of Medicine, Daegu, Korea

**Keywords:** small-molecule, neuroinflammation, PPAR-γ, microglia, astrocytes

Neuroinflammation is an important component of many neuroinflammatory and neurodegenerative diseases. Microglia and astrocytes play a key role in neuroinflammation. Activated glial cells under neuroinflammatory condition produce proinflammatory cytokines, chemokines, nitric oxide (NO), and other neurotoxic mediators. Thus, it has been conventionally thought that the inhibition of such glial activation might be an effective neuroprotective strategy under inflammatory or degenerative disease conditions in the central nervous system (CNS). However, recent studies indicated that activated glia could adopt either neuroprotective or neurotoxic phenotype depending on instigating stimuli present in the given microenvironment [1]. Lipopolysaccharide (LPS) and interferon-gamma are representative stimuli that induce neurotoxic phenotype, while interleukin-4 and interleukin-10 mainly induce neuroprotective one. Although it was initially believed that functional dichotomy such as M1 and M2 phenotypes exists representing two different functional phenotypes of microglia, it is now widely accepted that such classification is an oversimplified view, and that activated glia show a continuum of the two extreme functional phenotypes. Therefore, modulation of glial phenotypes, rather than mere blockade of glial activation, appears to be more desirable to regain the glial balance that was lost in various disease conditions. Especially, small-molecule-based approaches to switching glial phenotypes seem to be quite attractive, because small-molecule drugs have higher chances of crossing blood-brain barrier, thereby being effective in the CNS.

**Figure 1:**
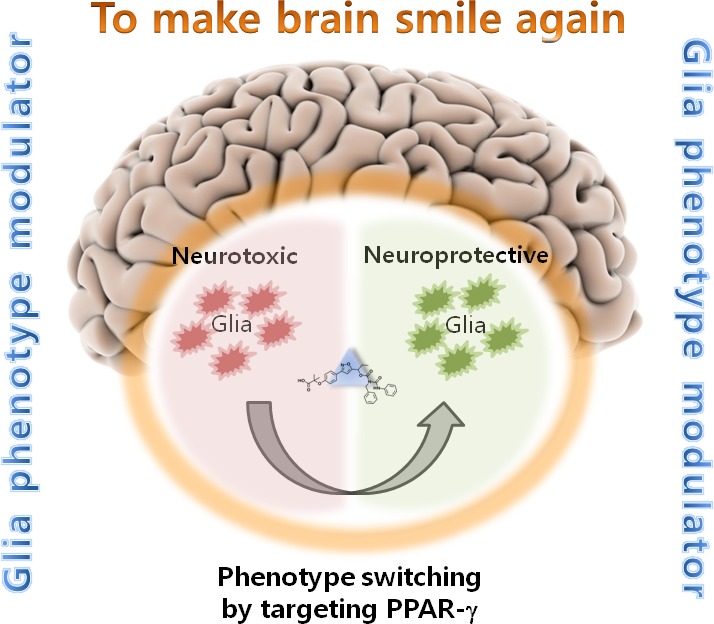
High-throughput screening identifies a glial phenotype modulator (GPM) that can make brain smile again The cell-based phenotypic screening was performed with 3,500 small-molecule compounds by measuring nitric oxide (NO) production in LPS-stimulated microglial cells. The screen identified a GPM compound with anti-inflammatory and phenotype switching effects on microglia and astrocytes. Further studies showed that PPAR-γ is the molecular target of the GPM. The GPM can be used to make sick brain smile by facilitating glial phenotype change from neurotoxic to neuroprotective state.

In a recent study by Song et al. [2], a phenotypic screen of small-molecule library using microglia cell-based assay was performed to search for glial phenotype modulators (GPM) (Figure 1). This screen identified a hit compound containing N-carbamoylated urethane moiety, which inhibited LPS-induced NO production in microglia. The small-molecule GPM compound inhibited proinflammatory cytokines and inducible nitric oxide synthase in LPS-stimulated microglia, and potentiated interleukin-4-induced arginase-1 expression, indicating that the GPM induces switching of microglial phenotype toward to an anti-inflammatory state. Peroxisome proliferator-activated receptor (PPAR)-γ was identified as a molecular target of the compound. The GPM specifically activated PPAR-γ, but not PPAR-δ or -α, in the PPAR response element reporter assay, thereby assuring that PPAR-γ is the authentic target protein of the GPM. Genetic and pharmacological inhibition of PPAR-γ attenuated phenotype switching effects of the GPM, further confirming the role of PPAR-γ as the molecular target of the GPM. When the study was extended to astrocytes, Song et al. found that the GPM similarly inhibited proinflammatory TNF-α and NO production, while enhancing the expression of anti-inflammatory genes, such as arginase-1 and Ym-1. These results suggested that the GPM induces an anti-inflammatory phenotypic switch in astrocytes as well. The effect of the GPM on glial activation was also tested in vivo using an LPS-induced mouse neuroinflammation model. In a pharmacokinetic study, the brain distribution of the GPM after intraperitoneal administration with relatively long half-life time was demonstrated. A quantitative analysis of the different brain regions revealed that the intraperitoneally administered GPM effectively blocked the systemic LPS-mediated microglial hyper-activation in cortex, hippocampus, and substantia nigra, suggesting a therapeutic potential for neuroinflammatory diseases.

In summary, based on a high-throughput screen of an approximately 3,500-member in-house library, Song et al. identified a novel small-molecule compound that has an anti-neuroinflammatory effect via the phenotypic switch towards the M2-like anti-inflammatory state in glia. The GPM is a specific PPAR-γ agonist that can be used to fine-tune glial phenotypes and functions. The GPM-mediated anti-inflammatory state of brain glia may lead to beneficial neuroprotective effects and has therapeutic potential in neuroinflammatory and neurodegenerative diseases. Recent studies suggested that functional phenotypes of macrophages and glia are closely associated with their glucose metabolism. A more recent study by Van den Bossche et al. [3] elegantly showed that NO produced under inflammatory condition inhibits M1 to M2 phenotype switch of macrophages by dampening mitochondrial respiration. They proposed that inhibiting NO production may improve mitochondrial function and reprograming towards M2-like phenotype. Thus, future investigation should look into optimal tactics that can modulate glial phenotypes from the metabolic perspectives.
